# Sofosbuvir in Combination with Simeprevir +/- Ribavirin in Genotype 4 Hepatitis C Patients with Advanced Fibrosis or Cirrhosis: A Real-World Experience from Belgium

**DOI:** 10.1371/journal.pone.0170933

**Published:** 2017-01-26

**Authors:** Delphine Degré, Thomas Sersté, Luc Lasser, Jean Delwaide, Peter Starkel, Wim Laleman, Philippe Langlet, Hendrik Reynaert, Stefan Bourgeois, Thomas Vanwolleghem, Sergio Negrin Dastis, Thierry Gustot, Anja Geerts, Christophe Van Steenkiste, Chantal de Galocsy, Antonia Lepida, Hans Orlent, Christophe Moreno

**Affiliations:** 1 Department of Gastroenterology, Hepatopancreatology and Digestive Oncology, CUB Hôpital Erasme, Université Libre de Bruxelles, Brussels, Belgium; 2 Department of Hepato-Gastroenterology, CHU Saint-Pierre, Brussels, Belgium; 3 Department of Gastroenterology, Brugmann Hospital, Brussels, Belgium; 4 Department of Hepatogastroenterology, CHU Sart Tilman, University of Liège, Liège, Belgium; 5 Department of Hepato-Gastroenterology, Clinique Universitaire Saint-Luc, Brussels, Belgium; 6 Department of Gastroenterology and Hepatology, University Hospital Gasthuisberg, Leuven, Belgium; 7 Department of Hepato-Gastroenterology, CHIREC, Brussels, Belgium; 8 Department of Gastroenterology and Hepatology, VUB, UZ Brussels, Brussels, Belgium; 9 Department of Gastroenterology and Hepatology, ZNA, Antwerp, Belgium; 10 Department of Gastroenterology and Hepatology, Antwerp University Hospital, Edegem, Belgium; 11 Laboratory of Gastroenterology and Hepatology, Department of Gastroenterology and Hepatology, Erasmus Medical Center, Rotterdam, The Netherlands; 12 Department of Gastroenterology and Hepatology, GHdC, Charleroi, Belgium; 13 Department of Gastroenterology and Hepatology, University Hospital Ghent, Ghent, Belgium; 14 Department of Gastroenterology and Hepatology, AZ Maria Middelares, Ghent, Belgium; 15 Department of Gastroenterology and Hepatology, Hôpitaux IRIS sud, Brussels, Belgium; 16 Department of Gastroenterology and Hepatology, AZ Bruges, Bruges, Belgium; University of North Carolina at Chapel Hill School of Dentistry, UNITED STATES

## Abstract

**Introduction:**

Hepatitis C virus (HCV) is a major global health issue and successful treatment has been associated with a reduction of risk of all-cause mortality. Advancements have been made in HCV treatment through the use of interferon-free regimens. Most trials have been conducted in HCV genotype (GT) 1 and data for interferon-free regimens in GT4 patients are limited. The aim of this study was to evaluate the safety and efficacy of sofosbuvir plus simeprevir in a real-world cohort of HCV GT4 patients with advanced fibrosis.

**Patients and Methods:**

Eighty-seven GT4 treatment-naïve or –Interferon (IFN) ribavirin (RBV) experienced patients treated with sofosbuvir and simeprevir +/- ribavirin (RBV) were enrolled in this cohort study (41% severe fibrosis, 59% cirrhosis).

**Results:**

Patients were 51.7% male, 78.2% IFN/RBV treatment-experienced, and 37.9% received RBV treatment. The overall sustained virologic response at least 12 weeks after treatment (SVR12) rate was 87.4% while patients treated with and without RBV had rates of 87.9% and 87% (p = 0.593), respectively, and patients with advanced fibrosis (F3) and patients with cirrhosis had SVR12 rates of 94.4% and 82.4% (p = 0.087), respectively. SVR12 rates in treatment-naïve patients and in IFN/RBV -experienced patients were 78.9% and 89.7% (p = 0.191), respectively. Treatment failure occurred most commonly in patients with cirrhosis and severe disease. The treatment was well tolerated and no patient died or discontinued treatment due to adverse events.

**Conclusions:**

Sofosbuvir in combination with simeprevir +/- ribavirin in GT 4 HCV patients with advanced fibrosis achieved high SVR12 rates and was well tolerated. RBV did not appear to increase the rate of SVR12.

## Introduction

Hepatitis C virus (HCV) is a major global health issue affecting 170 million patients worldwide [1]. Chronic infection leads to progressive liver fibrosis, end stage liver disease, or hepatocellular carcinoma [2,3] and successful treatment resulting in a sustained virologic response (SVR) has been associated with a reduction of risk of all-cause mortality in HCV patients [4,5]. Advancements have recently been made in HCV treatment through the use of interferon-free regimens that combine direct-acting antiviral agents (DAA), resulting in high efficacy rates and a better safety profile than those of interferon (IFN)-based regimens [6–10]. Most trials have been conducted in Western countries where HCV genotype (GT) 1 is prevalent. The phase 2 COSMOS study showed that combined simeprevir and sofosbuvir was efficacious and well tolerated in HCV GT1 infections [11]. Based on the results of this study, this treatment was recommended for GT1 HCV patients by the European Association for the study of the Liver (EASL) [12] and the American Association for the Study of the Liver (AASLD) [13]. The phase 3 OPTIMIST-1 study confirmed these results [14] and additional large, prospective cohort studies showed that combination of simeprevir plus sofosbuvir was associated with high rates of SVR and low rates of treatment discontinuation in real-world patients with HCV GT1 infection [15,16].

HCV GT 4 accounts for 20% of HCV infections worldwide [17] and is most prevalent in the Middle East and sub-Saharan Africa. In Egypt, GT4 accounts for approximately 90% of HCV infections [18] and its prevalence has increased in several European countries, which is considered to be a consequence of immigration [19–21]. The high genetic variability of HCV GT4 infection includes 17 confirmed subtypes, with subtype 4a predominately seen in Egypt, while Saudi Arabia and parts of Europe have high rates of subtypes 4a, 4c, and 4d [22,23]. Sofosbuvir or simeprevir in combination with pegylated IFN and ribavirin (RBV) improved SVR in GT4 patients [24–26]. However, efficacy and safety data regarding interferon-free regimens in HCV GT4 patients are currently scarce. Treatment with sofosbuvir and simeprevir was recommended in EASL clinical practice guidelines for HCV GT4 patients, but this recommendation was mainly based on the extrapolation of results in GT1 patients, since data with this combination in GT4 patients was limited [12]. In Belgium, sofosbuvir and simeprevir have been available since January 2015 for GT1 and GT4 patients with advanced fibrosis (F3-F4 METAVIR) for 12 weeks. The aim of this observational study was to evaluate the safety and the efficacy of this treatment in a real-world Belgian cohort of HCV GT4 patients with advanced fibrosis.

## Materials and Methods

### Patients

GT4- treatment naïve or –IFN/RBV experienced patients from 15 referral centers in Belgium treated with sofosbuvir and simeprevir +/- ribavirin were enrolled between January and September 2015 in this observational study. Reference centers allowed to prescribe DAAs were included in the study. All patients had advanced fibrosis (F3-F4) as required by national reimbursement criteria. Degree of fibrosis was defined at the beginning of treatment by histology or by combination of an elastography test and a biological fibrosis score with a maximum age of laboratory values of 1 year. The cut-offs for F3-F4 chronic HCV fibrosis assessment for transient elastography were for Fibroscan® (Echosens, Paris, France): F3 ≥9.5 kPA and F4 ≥12.5 kPA, and for shear wave elastography: F3 ≥8.7 kPA and F4 ≥10.4 kPA [27,28]. The cut-offs for biological fibrosis score for Fibrotest (Biopredictive) were F3:0.59–0.72, F3-F4:0.73–0.74, F4 ≥0.75, for APRI: F3≥1, F4≥1.6, for FIB-4: F3≥2.1, F3-F4≥3.25, and F4≥3.85 [29]. History of hepatic decompensation was defined by prior or current diagnosis of ascites, hepatic encephalopathy, spontaneous bacterial peritonitis or variceal hemorrhage. The study was performed after approval by the “comité d’éthique hospitalo-faculaire Erasme-ULB”: written informed consent was not required by the “comité d’éthique hospitalo-faculaire Erasme-ULB” because it was a retrospective, non-interventional study. Moreover, the majority of patients had sustained virological response after the treatment and was not followed regularly thereafter.

### Treatment

Sofosbuvir + simeprevir treatment was considered as standard of care treatment for participants as recommended by Belgian and EASL guidelines [12,30].The use of ribavirin (RBV) was at the discretion of the treating clinician. All but two patients received 12 weeks of treatment. The other two received 24 weeks of treatment. Patients were treated with a single 400 milligram tablet of sofosbuvir and a single 150 milligram tablet of simeprevir both taken orally daily. Ribavirin dosing was variable across patients but for the majority (24/33) of patients, ribavirin was administered according to body weight (<75 kg, 1000 mg/day and ≥75 kg, 1200 mg/day in 2 doses). Patients were followed up for at least 12 weeks after the end of treatment.

### Study assessments

Data collection included demographic data, history of previous HCV treatment, laboratory data including levels of serum alanine aminotransferase (ALT), aspartate aminotransferase (AST), total bilirubin, albumin, creatinine, hemoglobin, platelet count, International Normalized Ratio (INR), and HCV RNA. Severity of cirrhosis was assessed by Child-Pugh and MELD scores. These different parameters were also assessed at week 4 (W4) and week 12 (W12) of treatment when available.

Treatment efficacy was measured as sustained virologic response (SVR)12 defined as HCV RNA level below level of quantification at least 12 weeks after the end of treatment (EOT). For patients who did not achieve SVR12, the frequency of relapse, and non-response was reported. The numbers of patient lost-to-follow-up was also reported.

### Statistical analysis

The primary endpoint was SVR12. Other efficacy endpoints were virological response at W4 and HCV RNA at the end of treatment. The primary analysis was done in the intention-to-treat (ITT) population including all patients who received at least one dose of treatment. An additional analysis of the primary endpoint was performed that excluded patients who did not have SVR12 values due to missing data, or lost to follow-up. The demographic data were compared between groups using Student’s t-test or the Mann-Whitney U test as appropriate. Categorical variables were studied using the two-sided Chi-square test. A p value <0.05 was considered statistically significant. Calculations were performed with SPSS 18.0 software (Chicago, IL, USA).

## Results

### Patient population

Eighty-seven patients with HCV GT 4 were included between January 2015 and September 2015. Thirty-six patients (41.4%) had severe fibrosis (F3) and 51 (58.6%) were patients with cirrhosis. The study population included 46 (51.7%) male and 68 (78.2%) IFN/RBV-experienced patients. The majority of patients were black Africans (58.6%). Median age was 61 (30–81) years and 9 patients were HCV/HIV co-infected. Thirty-three (37.9%) patients received ribavirin treatment, 12 (33%) of patients with advanced fibrosis and 21 (41.2%) of patients with cirrhosis. In patients with cirrhosis, median MELD score and Child-Pugh score were 8 (6–19) (range) and 5 (5–9) (range), respectively, 43.7% patients had platelet counts below 100 000/mm³, 28.2% had albumin <35g/L and 13 patients had a history of liver decompensation. At baseline, the clinical and demographic characteristics of patients who received ribavirin were similar to those who did not receive ribavirin, except for ALT levels which were higher in patients treated with ribavirin. The majority of patients received 12 weeks of treatment. Demographic and baseline characteristics are shown in Table 1.

**Table 1:** Characteristics of genotype 4 hepatitis C patients treated with simeprevir plus sofosbuvir with or without ribavirin.

Characteristic	Overall (n = 87)	SOF/SMV (n = 54)	SOF/SMV/RBV (n = 33)	p
Median age, years (range)	61 (30–81)	61.5 (30–81)	58 (40–76)	0.381
Male sex, n/N (%)	46/87(52.9)	26/54(48.1)	20/33(60.6)	0.182
IFN/RBV-experienced patients, n/N(%)	68/87(78.2)	39/54(72.2)	29/33(87.9)	0.071
F3, n/N(%)	36/87(41.4)	24/54(44.4)	12/33(36.4)	0.303
F4, n/N(%)	51/87(58.6)	30/54(55.6)	21/33(63.6)	
Ethnicity, n/N(%)				0.621
Black African	51/87(58.7)	34/54(63)	17/33(51.6)	
Caucasian	26/87(29.9)	15/54(27.8)	11/33(33.3)	
Middle East	8/87(9.2)	4/54(7.4)	4/33(12.1)	
Maghrebin	1/87(1.1)	1/54(1.8)	0/33(0)	
Unknown	1/87(1.1)	0/54(0)	1/33(3)	
GT4 subtypes, n/N				
4a	2/87			
4e	6/87			
4h	3/87			
4acd	5/87			
unknown	71/87			
Median ALT, IU /mL (range)	67 (12–505)	57 (12–327)	80 (24–505)	0.01
Median bilirubin, mg/mL (range)	0.7 (0.1–5.7)	0.7 (0.2–5)	0.8 (0.1–5.7)	0.389
Median INR	1.1 (0.9–2.2)	1.1 (0.9–2.2)	1.1 (1–1.6)	0.808
Median platelet count, 10³/mm³ (range)	131 (23–320)	135.5 (23–320)	118 (30–276)	0.277
Albumin, g/L (range)	40 (24.5–49)	40 (28–49)	40 (24.5–47.1)	0.472
Ascites (cirrhosis), n/N(%)	2/47(4.3)	2/28 (7.1)	0/19 (0)	0.350
Encephalopathy (cirrhosis), n/N(%)	1/46 (2.2)	0/27(0)	1/19(5.3)	0.413
MELD(range) (patients with cirrhosis)	8 (6–19)	8 (6–19)	9 (7–16)	0.341
Child-Pugh(range) (patients with cirrhosis)	5 (5–9)	5 (5–9)	5 (5–8)	0.670
Treatment duration				0.141
12W, n/N(%)	85/87(97.7)	54/54(100)	31/33(93.9)	
24W, n/N(%)	2/87(2.3)	0/54(0)	2/33(6.1)	

### Efficacy

**Fig 1:**
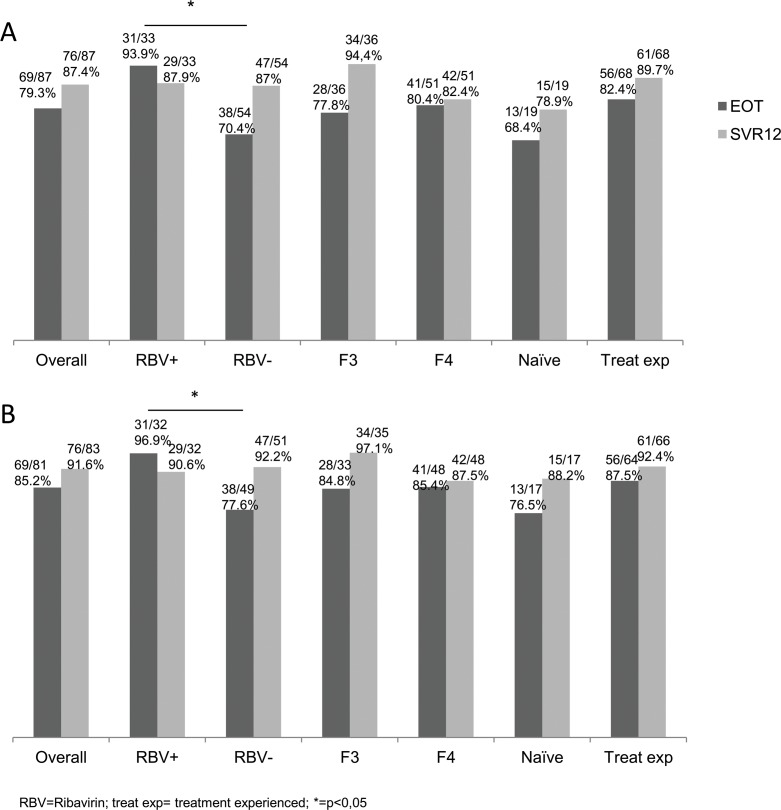
Patients who achieved sustained virological response 12 weeks after the end of treatment (A) In the intention to treat population according to ribavirin treatment, degree of fibrosis, and treatment history. (B) In a sensitivity analysis that excluded patients in whom HCV viral load at the end of treatment or 12 weeks post treatment were missing.

In an ITT analysis, SVR12 rate was 87.4% (76/87). Among the patients who failed to achieve SVR12, 5 had post treatment relapse, 1 patient had lack of efficacy during treatment with detectable HCV RNA but below lower limit of quantification (LLOQ) at the end of treatment and a relapse after, 1 patient was lost to follow up during the treatment but had a positive HCV viral load after the end of treatment (no compliance data is available for this patient) and 4 patients were lost to follow-up after completing treatment. SVR12 in patients treated with and without RBV were 87.9% (29/33) and 87% (47/54) (p = 0.593), respectively, and among patients with cirrhosis, SVR12 in patients treated with and without RBV were 81% (17/21) and 83.3%(25/30) (p = 0.555), respectively. SVR12 in patients with advanced fibrosis (F3) and in patients with cirrhosis were 94.4% (34/36) and 82.4% (42/51) (p = 0.087), respectively. Moreover, SVR12 in treatment-naïve patients and in IFN/RBV-experienced patients were 78.9% (15/19) and 89.7% (61/68) (p = 0.191), respectively. HCV RNA at the end of treatment was undetectable in 69 patients (79.3%). HCV RNA was detectable but below LLOQ in 12 patients and data were missing for 6. However, among the 12 patients with detectable HCV RNA but below LLOQ at the EOT, 10 achieved SVR12 (1 failure of treatment and 1 lost to follow-up). The number of patients with undetectable HCV RNA at the EOT was higher in patients treated with RBV (93.9%) than in patients without RBV (70.4%) (p = 0.007) (Fig 1A). An additional analysis that excluded patients who did not achieve SVR12 for reasons other than virologic failure (missing SVR12 data for 4 patients lost to follow-up) demonstrated an SVR12 rate of 91.6% (76/83) (Fig 1B). The characteristics of patients who failed to achieve SVR12 are shown in Table 2. All but one patient who failed to achieve SVR12 were patients with advanced cirrhosis with high MELD score or low platelet count or low albumin level. Only one patient was not a patient with cirrhosis but this patient was lost to follow-up during the treatment and no compliance data was available for this patient.

**Table 2:** Characteristics of patients who failed to achieved SVR12.

	Patient 1	Patient 2	Patient 3	Patient 4	Patient 5	Patient 6	Patient 7
Fibrosis	F4	F4	F4	F4	F4	F4	F3
Ribavirin	No	Yes	Yes	Yes	No	No	Yes
Treatment-experienced	No	Yes	Yes	Yes	Yes	Yes	Yes
HIV co-infection	No	No	No	No	No	No	No
Ethnicity	Caucasian	Black African	Caucasian	Caucasian	Black African	Black African	Caucasian
Age	41	66	48	56	49	69	59
Gender	Male	Female	Male	Male	Female	Male	Male
Baseline MELD score	19	15	13	13	11	9	Not applicable
Baseline Child-Pugh score	9		8	8	5	7	Not applicable
Platelet count, 10³/mm³	100	50	30	42	35	61	Not reported
Albumin, g/L	28		30	24	36	28	Not reported
Baseline HCV RNA	Positive	4.87	6.1	6.36	6.96	6.39 log	6.44
W4 HCV RNA	31	Not detected	51	Not detected	383		Not reported
EOT HCV RNA	Not detected	Not detected	Not detected	Not detected	Not detected	Weakly positive	Not reported
PTW12 HCV RNA	177932	5618	432140	213796	44530000	Positive	2404200

Finally, HCV RNA was measured at week 4 of treatment in 67 patients. HCV viral load was undetected in 22 patients (32.8%), 14/28 (50%) in patients treated with RBV and 8/39 (20.5%) in patients without RBV (p = 0.012). There was no SVR12 rate difference between patients with undetectable HCV RNA at W4 (20/22; 90.9%) and patients with positive HCV RNA at W4 (42/45; 93.3%) (p = 0.534).

### Safety and tolerability

During the treatment, SAE were observed in 3 patients. One patient was hospitalized for anemia due to rectal blood loss resolved after transfusion and not related to the treatment, 1 patient was hospitalized for encephalopathy not considered to be related to the treatment and 1 patient developed hepatorenal syndrome possibly related to the treatment. No patient died or discontinued treatment due to adverse events. Among patients treated with and without ribavirin, 5/33 (15.2%) and 3/51 (5.9%) had hemoglobin<10g/dl (p = 0.132) during the treatment and ribavirin dose was decreased in 3 of the 33 RBV-treated patients and stopped in 3 patients. The 6 patients who modified RBV dosage during treatment all had cirrhosis. The median baseline haemoglobin level for the 6 patients who modified their ribavirin dosage was 13.5 [11.5–15.3] g/dL compared with 14.4 [11.5–17.7] g/dL for the 27 patients who had no modifications to their ribavirin dosage during the study (p = 0.190) and the median baseline RBV dosage was 900 [800–1200] mg in patients who modified RBV dosage and 1200 [400–1200] mg in patients without modification of RBV dosage (p = 0.189).

## Discussion

In this Belgian real-world cohort of HCV GT4 patients with advanced fibrosis, we showed that the combination of SOF + SMV achieved in ITT analysis an SVR12 rate of 87.4%, 87.9% for patients treated with RBV and 87% for patients treated without RBV. After exclusion of patients who did not achieve SVR12 for reasons other than virologic failure, we observed an SVR rate of 91.6%. These results showed that the combination of SOF+SMV is efficacious in HCV GT4 patients with advanced fibrosis and that the addition of RBV did not appear to increase the rate of SVR12 although patients treated with RBV seemed to have a faster decrease of their viral load with a higher rate of negative viral load at week 4 and at the EOT. Interestingly, some patients had detectable HCV RNA but below LLOQ at the EOT but achieved SVR12. We did not observe a significant difference in SVR12 between patients with severe fibrosis F3 and patients with cirrhosis. This may be due to the small size of the cohort. However, all but one patient who failed to achieve SVR12 were patients with cirrhosis and advanced disease. This observation is consistent with results observed previously in other studies [15]. After exclusion of patients who did not achieve SVR12 for reasons other than virologic failure, we observed an SVR12 rate of 87.5% in patients with cirrhosis. This result is comparable to the result of the OPTIMIST-2 study in GT1 patients with cirrhosis treated with SOF + SMV, who achieved SVR12 of 83% [31] while decompensated patients were excluded in this study. One patient with positive viral load after the end of treatment was a patient without cirrhosis but this patient was lost to follow-up during the treatment and no compliance data was available. Finally, SVR12 rate in IFN/RBV experienced patients and treatment naïve patients was not significantly different. Treatment was well tolerated. No patient died or discontinued treatment due to adverse events. Among patients treated with RBV, 18% of patients modified ribavirin dosage. The median baseline hemoglobin levels and the median baseline RBV dosage did not differ between patients who modified ribavirin dosage compared with those patients who did not. However, RBV posology was decreased only in patients with cirrhosis.

Limited data are currently available to guide treatment in chronic HCV GT4 patients especially with advanced fibrosis. However it is important to develop optimal treatment strategies for HCV GT4 patients because this genotype is highly endemic in non-Western parts of the world and its prevalence has increased in several European countries [19–21] Moreover, in real life, patients with advanced fibrosis have an urgent need of treatment and robust data are lacking for this population. Several treatment regimens containing sofosbuvir or simeprevir have been evaluated previously for GT4 HCV patients. The combination of SOF and RBV for 24 weeks in a cohort of Egyptian patients showed an SVR rate of 90% but this cohort included few patients with cirrhosis and the SVR rate in patients with cirrhosis was lower (78%) [32]. Recently, a real life study including HCV GT4 patients with advanced fibrosis showed that SOF/SMV+/- RBV combination for 12 weeks was an effective regimen with an overall SVR rate of 92% [33].Other studies including a few patients with cirrhosis showed that sofosbuvir with ledipasvir [34] had also high SVR rates in GT4 patients. A recent study evaluated the efficacy of the SOF+ ledipasvir+ RBV combination in GT1 and GT4 patients with cirrhosis and seemed to be promising but the number of GT4 patients was low[35]. Other studies have evaluated the efficacy of newer direct-acting antiviral therapies for treatment of HCV GT4 patients including the combination of grazoprevir and elbasvir [36]. This treatment seemed to be efficacious but the results in patients with cirrhosis seemed to be worse [37]. The combination of sofosbuvir and velpatasvir provided high rates of SVR among patients infected with HCV genotypes 1,2,4,5, and 6, including those with compensated cirrhosis [38] and decompensated cirrhosis [39]. However, this study included few GT 4 patients. The AGATE-1 study evaluated the efficacy of the combination of ombitasvir and paritaprevir/ritonavir with ribavirin in HCV GT4 patients with cirrhosis. This study showed that this regimen is very efficacious for GT4 patients with cirrhosis [40]. However, conversely to the SOF-SMV combination, RBV use is obligatory. Moreover, this combination is not allowed in patients with decompensed cirrhosis. Indeed, post-marketing surveillance identified several cirrhotic patients who developed hepatic decompensation or liver failure while receiving this therapy [41].

Our study is subject to several limitations. First, ribavirin treatment was not given after randomization but only at the discretion of the treating clinician and the number of patients with cirrhosis receiving RBV is probably too small to show a potential benefit of RBV treatment in this population. It is thus difficult to make definitive conclusions concerning the role of ribavirin in the efficacy of treatment in our cohort of patients. Moreover, we had no data about resistance associated variants (RAV) which might influence the treatment response. Indeed, in the COSMOS study, the viral relapse rate was mainly correlated with mutations that have previously been associated with simeprevir resistance [11]. However, the Q80K mutation has not been reported in GT4.

In conclusion, we showed in this real-world cohort of GT4 patients with severe fibrosis and cirrhosis that the combination of SOF and SMV is efficacious and well tolerated and represents a good therapeutic option in HCV GT4 patients with advanced fibrosis and compensated cirrhosis. In patients with decompensated cirrhosis, second-generation IFN-free combinations would be better suited.
